# Preparing Excitable Cardiac Papillary Muscle and Cardiac Slices for Functional Analyses

**DOI:** 10.3389/fphys.2022.817205

**Published:** 2022-03-03

**Authors:** Bradley M. Palmer, Stephen P. Bell

**Affiliations:** ^1^Department of Molecular Physiology and Biophysics, University of Vermont, Burlington, VT, United States; ^2^Department of Medicine, University of Vermont, Burlington, VT, United States

**Keywords:** cardiac, excitable, force, slice, papillary, muscle

## Abstract

While the reductionist approach has been fruitful in understanding the molecular basis of muscle function, intact excitable muscle preparations are still important as experimental model systems. We present here methods that are useful for preparing cardiac papillary muscle and cardiac slices, which represent macroscopic experimental model systems with fully intact intercellular and intracellular structures. The maintenance of these *in vivo* structures for experimentation *in vitro* have made these model systems especially useful for testing the functional effects of protein mutations and pharmaceutical candidates. We provide solutions recipes for dissection and recording, instructions for removing and preparing the cardiac papillary muscles, as well as instruction for preparing cardiac slices. These instructions are suitable for beginning experimentalists but may be useful for veteran muscle physiologists hoping to reacquaint themselves with macroscopic functional analyses.

## Introduction

The middle of the last century witnessed a dramatic turn in the muscle physiology field toward the molecular. A recent historical review suggests that the early 1970s marks a time when protein biochemistry of muscle contraction overtook the previously conventional approaches to understanding muscle function (Szent-Györgyi, 2004). The field at the molecular level has advanced tremendously since then, especially with the advent of molecular genetics techniques. With these new tools, select mutations in muscle proteins could be produced, and the functional consequences at the molecular level assessed. In this way, the molecular basis of muscle function could be examined one protein at a time and with the precision of one amino acid at a time.

Yet inferring macroscopic muscle performance from molecular-level results is not always straightforward. For example, a single point mutation in cardiac myosin known to underlie the development of a cardiomyopathy can cause little to no observed effect in molecular function, and the result would often go unpublished. Perhaps more confusing are those cases when a mutation in a seemingly ancillary muscle protein, for example, muscle LIM protein in the sarcomeric Z-disk, can cause a significant cardiomyopathy (Morita et al., 2005). These examples serve to support the importance of examining intact muscle function even in the age of molecular manipulations. Hence the value of transgenic mouse models, which allowed the examination of protein mutations in the context of an intact working muscle (Fatkin et al., 1999).

The examination of the cardiac slice has recently gained deserved attention due to the ease with which a slice can be generated and maintained (Pitoulis et al., 2020). Cardiac slices can remain viable for weeks and even months (Watson et al., 2017; Fischer et al., 2019). With this muscle model system, viral transfections of proteins are possible thus minimizing the developmental of compensatory secondary effects that mutations may cause in the transgenic animals (Moretti et al., 2020). For this and many other reasons, the cardiac slice is likely to become an important model system for examining macroscopic muscle function due to molecular-level manipulations including response of pharmaceuticals.

This methods paper describes how to prepare a cardiac papillary muscle and cardiac slice for examination with a conventional force transducer and length controller. We provide details related to the instruments required that would allow a beginning muscle experimentalist or a starting laboratory to prepare these experimental model systems. We do not provide details related the apparatus used to examine muscle function nor details related to specific protocols to examine muscle function. The procedures provided here are illustrated using rat heart but are also applicable to other species, such as mouse, guinea pig, ferret, and rabbit, and other muscle types, such as cardiac trabeculae, cardiac strips prepared from endo- or epicardium, and skeletal muscles with intact tendons.

## Materials and Methods

### Papillary Muscle Preparation

We will not describe how to anesthetize the rat and remove the heart. Please seek out instruction from your institutional veterinarian. All procedures and use of animals described here adhere to the Guidelines of the American Physiological Society and have been reviewed and approved by the Institutional Animal Care and Use Committee of the University of Vermont. A video demonstration of dissecting the left ventricle and removing the papillary muscles can be found at this link.^1^

Have ready a small beaker (~50 ml) filled about halfway with Dissection Solution chilled to 0–4°C. Also have ready a small shallow glass container with a silicon bottom also filled about halfway with Dissection Solution and sitting on the cooling plate. Make sure the solutions are being bubbled with 100% O_2_.

After the heart has been removed, place the heart into the small beaker. Using forceps, hold the atria and vessels above the base of the heart and shake the ventricles in the beaker to wash away as much blood as possible. Then place the heart into the shallow dish on the cooling plate. Removing and transferring the heart from the animal to the small beaker and then to the shallow dish on the cooling plate should be done quickly but without rushing and take under 1 min.

#### Exposing the Endocardium

**Figure 1:**
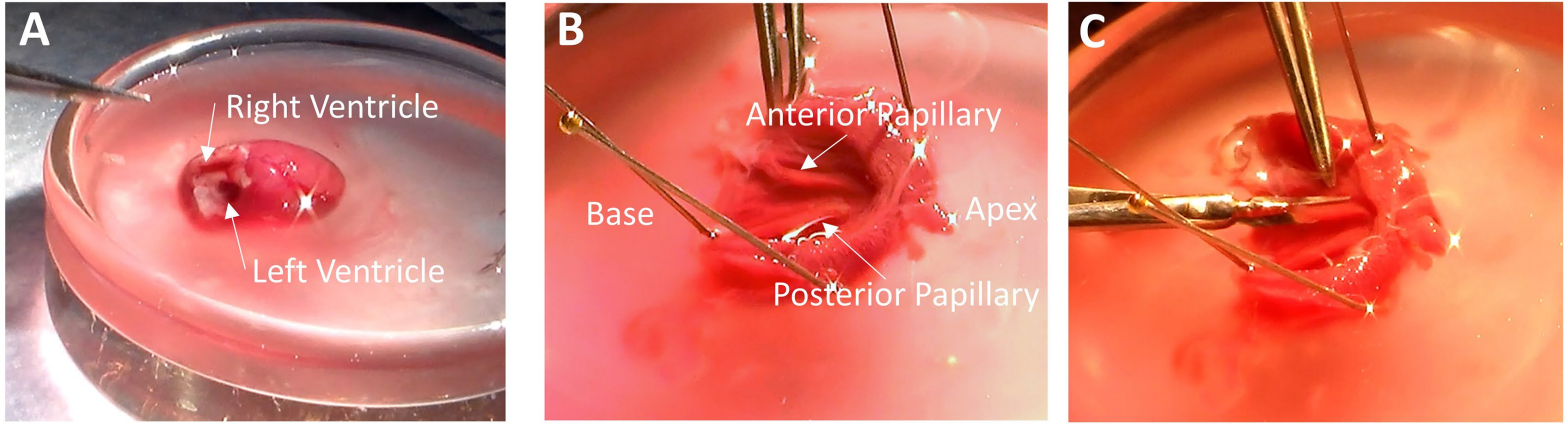
Removing Papillary Muscles. **(A)** With the atria trimmed away, the base of the heart is exposed, and the ventricles can be easily identified. The right ventricular cavity is crescent-shaped, and its free wall is thin. The left ventricular cavity is more circular in shape, and the walls are thick. **(B)** After removing the right ventricle and cutting down the septum, the endocardium is exposed with the anterior and posterior papillary muscles visible. **(C)** Remove the papillary muscles by cutting gently along the length of the muscle while being sure not to pull on muscle with the forceps.

The following sections are facilitated by use of the dissection microscope. Nevertheless, many experimentalists can expose the endocardium without use of the microscope. It is important that the tasks are performed accurately but also as quickly as possible without rushing. Using a pair of forceps and a pair of scissors, trim away the atria, valves and blood vessels that lie above the base of the ventricles. Once the atria have been trimmed away, the openings to the right and left ventricles can be seen at the base of the heart. If there is any blood obscuring your view, use transfer pipettes to remove blood and refill the volume with fresh dissection solution. The right ventricle can be identified by its thin wall, and it is opening at the base is crescent-shaped (Figure 1A). The opening of the left ventricle is circular and surrounded by thick muscular walls (Figure 1A). Removing the atria and exposing the base of the heart should be done in under 1 min after the heart is placed in the shallow dish.

Using forceps, hold the free wall of the right ventricle and trim it away to expose the septum. Now hold the remaining left ventricle with the septum pointing up. Place a scissors blade (~10–20 mm long) into the left ventricular cavity and cut down the center of the septum from the base to the apex. Cutting down the center of the septum will assure that the papillary muscles are not damaged by the scissors. Removing the right ventricular free wall and cutting down the septum should be done in under 2 min.

#### Removing the Papillary Muscles

Taking a few dissection pins, pin down both sides of the now cut septum to expose the endocardium. It is sometimes useful to pin down the apex, too, although in this photo the apex has been cut open prior to pinning. In Figure 1B, the heart is oriented with the apex to the right and base to the left. The anterior and posterior papillary muscles are now visible. In this orientation, the anterior papillary muscle is at the top of the image, and the posterior papillary muscle, which is typically thicker than the anterior papillary muscle, is at the bottom.

Now cut out the papillary muscles using a finer pair of scissors with a ~2–4 mm blade. It is very important not to hold the muscles with the forceps. Pinching or pulling the muscle will damage it and render it useless for later physiological analysis. Cut along the length of the papillary muscle to release it from the rest of the endocardium (Figure 1C). Once the papillary muscles have been removed, place them into another glass or clear plastic container holding the omega clips, looped suture, and bubbled dissection solution. Removing the papillary muscles should require 2-4 min.

#### Clipping a Papillary Muscle

**Figure 2:**
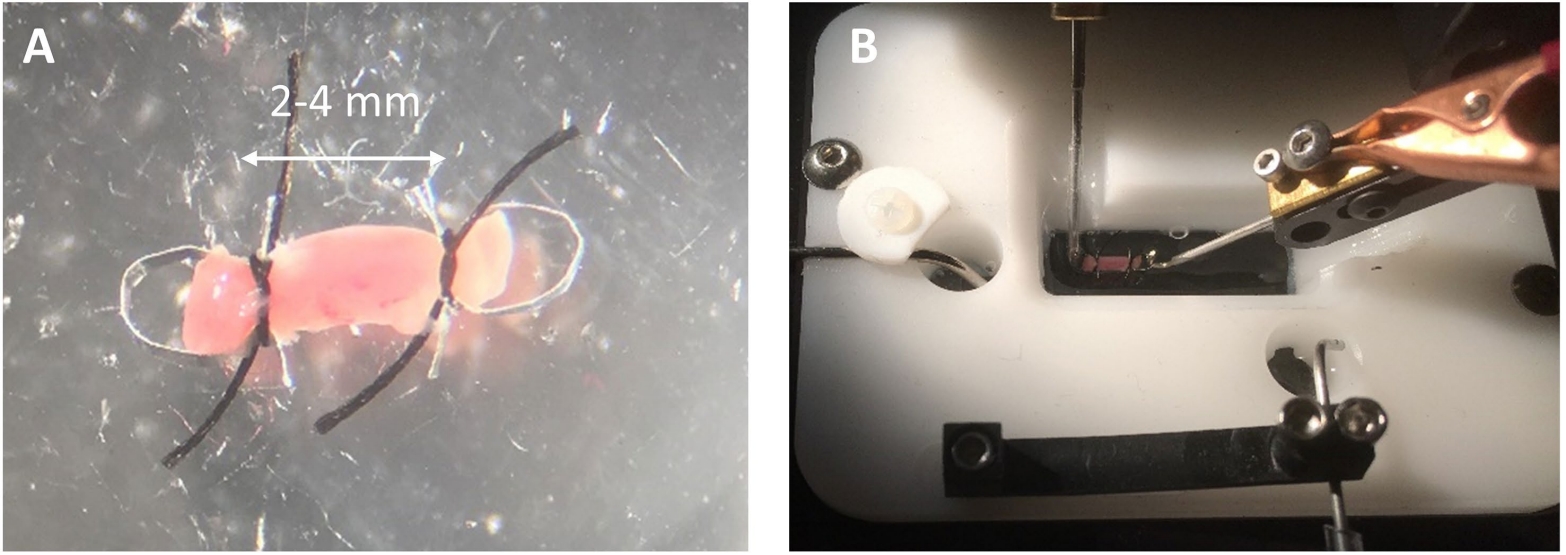
Clipping and Mounting Papillary Muscle. **(A)** A clipped rat cardiac papillary muscle is about 2–4 mm long. **(B)** The omega clips are placed over the hooks of the measurement apparatus and perfused with solution.

Trim away any excess endocardium from the papillary muscle being careful to grab only the tissue to be cut away and not to grab the papillary muscle with the forceps. Trimming does not need to be perfect, but good enough to assure a reasonably cylindrical muscle. Place the muscle in the dish with its long axis running back and forth away from your position. Bring an omega clip and a suture loop into the field. The resting distance between the feet should be narrower than the diameter of the muscle. Using two forceps, gently pull apart the feet of the omega clips and then placed the feet over the end of the muscle. Upon letting go of the feet, the spring action of the omega clip will recoil and maintain its position on the muscle during suturing. Grab one foot of the omega clip and lift it off the bottom and slip the loop over the head of the omega clip. Pull on the ends of the suture so the knot is tightened near the feet of the omega clip (Figure 2A). It is important that the suture be tight, but not over-tightened, which can cut into the muscle. The excess suture is then cut away. Repeat the process for the other end of the muscle. A video demonstration of clipping and mounting the papillary muscles can be found at this link.^2^

Note that, by the time the muscle is clipped, only the muscle between the clips will be characterized for function. The muscle at the point of the clipping, that is, between the feet, is considered connective. Whatever damage may occur to muscle function between the feet should not affect the recorded muscle function unless the damage includes cutting of the muscle.

#### Mounting a Papillary Muscle

Carry the small dish with the clipped muscle to the chamber. Adjust the distance between the platinum hooks using the manual micromanipulator so the hooks are slightly closer together than the distance between the two heads of the omega clips. Make sure the chamber is already filled with Recording Solution. Using forceps, pick up the muscle by a suture and carry it to the chamber. Place one omega clip over one hook and then place the other omega clip over the other hook (Figure 2B). The reader may wish to skip ahead to section “Preparing to Record Functional Data” to continue with recording force from a papillary muscle.

### Cardiac Slice Preparation

The preparation of a cardiac slice begins with exposing the endocardium. The methods outlined above for exposing the endocardium should be followed. It is also possible to remove the papillary muscles and use the remaining left ventricle for the preparation of the cardiac slice. A video demonstration of preparing, clipping, and mounting a cardiac slice can be found at this link.^3^

#### Making a Cardiac Slice

An excellent description of cardiac slice preparation can be found in Watson et al. (2017), which provides a detailed account of reagents, procedures, and expectations. A vibratome is used to slice thin layers of the cardiac tissue. The vibratome will vibrate its blade back and forth while traveling forward and slicing the tissue. It is important that the vibratome allow for adjustments to vibration amplitude and speed of travel, whose values will be addressed below. Standard safety razors are adequately sharp for cutting the tissue.

**Figure 3:**
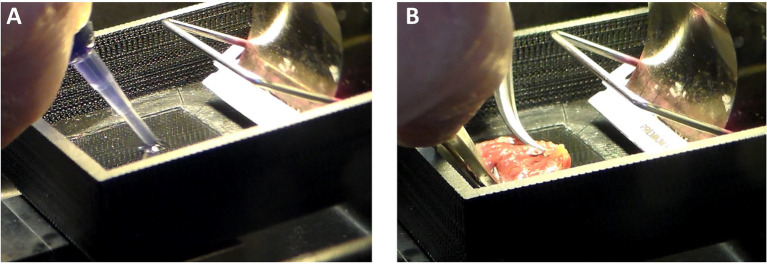
Cardiac Slice Preparation. **(A)** A cyanoacrylate glue (Histoacryl, Braun) is applied to the stage on which the heart will adhere. **(B)** The epicardium is pressed against the glue to leave the endocardium exposed for slicing with the vibratome.

There must be a platform or stage for the heart to be glued down. In Figure 3A, the stage is a glass slide about 45 mm long and 25 mm wide. This size stage is large enough for hearts from small mammals (up to size of a rabbit), but a larger stage and blade would be necessary if slicing hearts or larger heart sections from pigs or humans. A thin layer of a cyanoacrylate adhesive (Histoacryl, Braun) is applied to the glass. The left ventricle, which has already been cut down its septum, is then dabbed onto paper or gauze to remove excess solution from the epicardium. The tissue must still be wet, but not dripping with solution. The left ventricle is then laid onto the glue and pressed down with forceps for about 20–40 s (Figure 3B), after which the epicardium is glued to the glass and the endocardium exposed. Note that, while the left ventricle has been kept cold, the glass slide is not chilled at this point. We have found the glue is less adherent if the glass is chilled prior to forming a seal between tissue and glass.

Once glued down, put cold and oxygenated Dissection Solution onto the tissue with a transfer pipette. As solution starts to fill the chamber, excess glue will tend to float. Use a transfer pipette to remove the excess glue from the solution and continue to fill the chamber with fresh solution. Once the chamber is about half filled, ice or iced water can be placed around the stage. Be careful that ice does not get into the Dissection Solution bathing the tissue. To maintain viability, bubble with 100% O_2_.

The height of the blade should now be adjusted to assure that the blade will cut 0.5–1 mm below the surface of the endocardium. Turn on the vibratome, and the blade will slowly move across the tissue while vibrating. The settings for each vibratome and each laboratory will vary. Reasonable settings should include a relatively high amplitude of vibration and slow speed of travel. We typically use a vibration amplitude of ~1 mm peak to peak with vibration frequency ~200 Hz and travel speed ~5 mm per min or slower. With such slow travel, cutting one slice will take several minutes.

The first slice is usually not useable, as it will retain the uneven surface of the endocardium. The blade is then retracted. If the slice is still tethered to the left ventricle, remove the tethers with small scissors and remove the slice with forceps being careful not to pull on the tissue. The relative blade height is then adjusted by raising the tissue stage a distance that will define the thickness of the next slice. Typically, the thickness of each slice is between 0.2 and 0.4 mm. The next slice or two may also be unusable due to uneven surfaces. When the cut exposes a flat surface, the subsequent slices are useable. Place the slices into cold, oxygenated Dissection Solution. The creation of 3–5 slices can take approximately 30 min depending on the size of the heart and the cutting speed chosen. The yield from a single rat heart is typically on the order of 3–10 intact useable cardiac slices depending on the size of the heart and the thickness of the slices.

#### Short Term Viability of a Cardiac Slice

**Figure 4:**
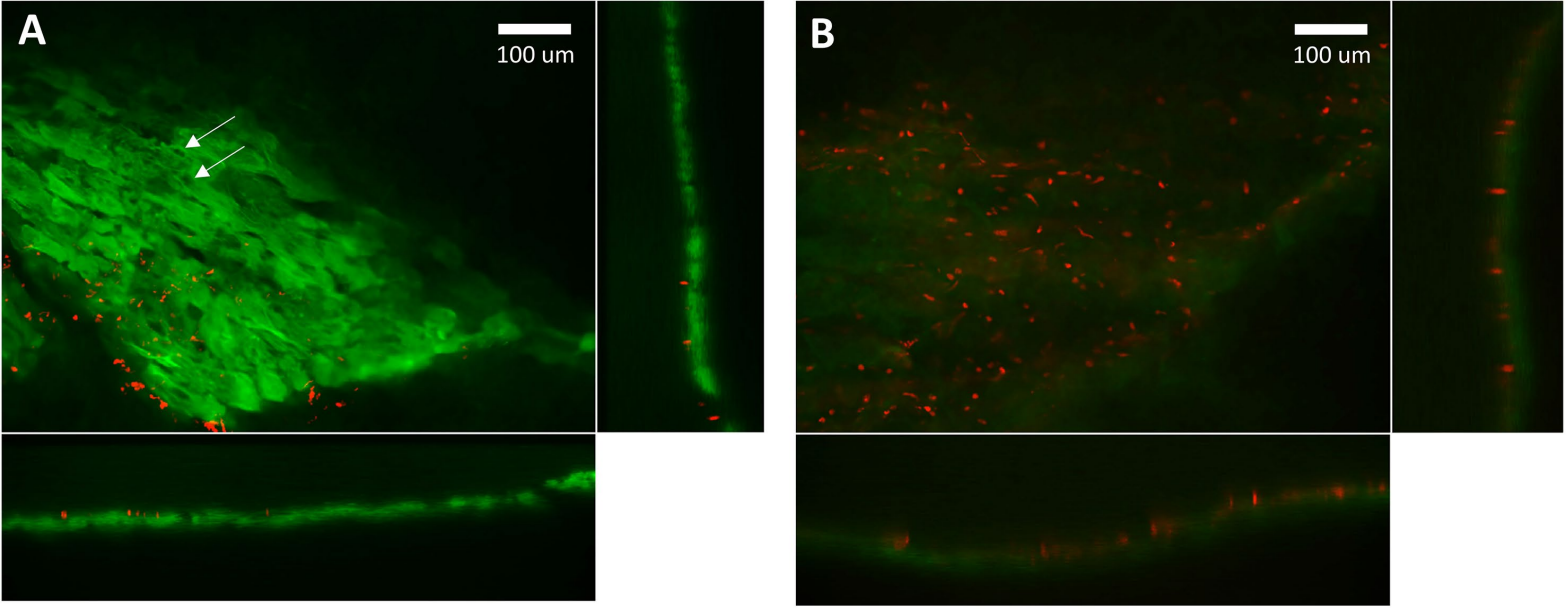
Confocal microscopy imaging of cell viability after slicing. **(A)** Green indicates cytosol of intact myocytes within a cardiac slice. Red indicates detection of DNA, which is only possible if the membrane has been disrupted. The localized absence of green within a myocyte indicates intact nuclei (arrows). DNA fragments and disrupted membranes appear limited to the edge of the slice where a razor blade was used to cut the tissue. We estimate 40% intact myocytes on the edges and effectively 100% intact for myocytes further than 30 mm from an edge. **(B)** Red indicates DNA detected in “dead” myocytes whose membranes have been disrupted by detergent. The absence of green further attests to a lack of intact membranes. Settings used for acquisition and display of these two images were the same.

We examined the viability of myocytes within cardiac slices with Thermo Fisher Scientific LIVE/DEAD Cell Imaging Kit 488/570, which uses 488 nm and 570 nm excitation wavelengths to excite calcein with emission at 510 nm and bobo-3 with emission at 605 nm. The calcein channel, displayed in green, indicates live intact myocytes, and the bobo-3 channel, displayed in red, indicates DNA and DNA fragments. Figure 4A demonstrates a high fraction of intact myocytes, whose cytosol volumes are shown in green, for myocytes away from the slice edge. A smattering of DNA or DNA fragments, shown in red, are apparent near the edges. Figure 4B demonstrates the expected image for myocytes that have been disrupted, in this case by the detergent Triton X-100 at 0.1% concentration by volume exposed for 30 min at room temperature. We estimate the fraction of intact viable myocytes along an edge to be 40% upon examining tissue with 20 μm depth from the edge, and the fraction of intact myocytes away from the edge is effectively 100%. For a typical slice 300 μm thick and 8 mm wide, total cross-sectional area is 2.4 mm^2^ with 0.33 mm^2^ attributed to edges and 2.07 mm^2^ away from edges. The net viability of myocytes within a typical slice was therefore estimated at 92%, which is consistent with the estimate reported by Watson et al. (2017). The effective cross-sectional area, which is necessary for normalizing force to calculate tension, is therefore 2.2 mm^2^ for this example.

We have found that cardiac slices can be maintained on a cooling plate at 0–4°C and bubbled with 100% O_2_ for up to 24 h. The long-term maintenance of cardiac slices over days, weeks, and months requires equipment, strategies, and techniques not described here and is beyond the scope of this paper.

#### Clipping a Cardiac Slice

The small, triangularly shaped clips are required to attach the slice to the recording apparatus. We use laser cut acrylic 0.5 mm thick and 10 mm wide at its broadest, but the clips can also be 3D printed and made of various plastics.

**Figure 5:**
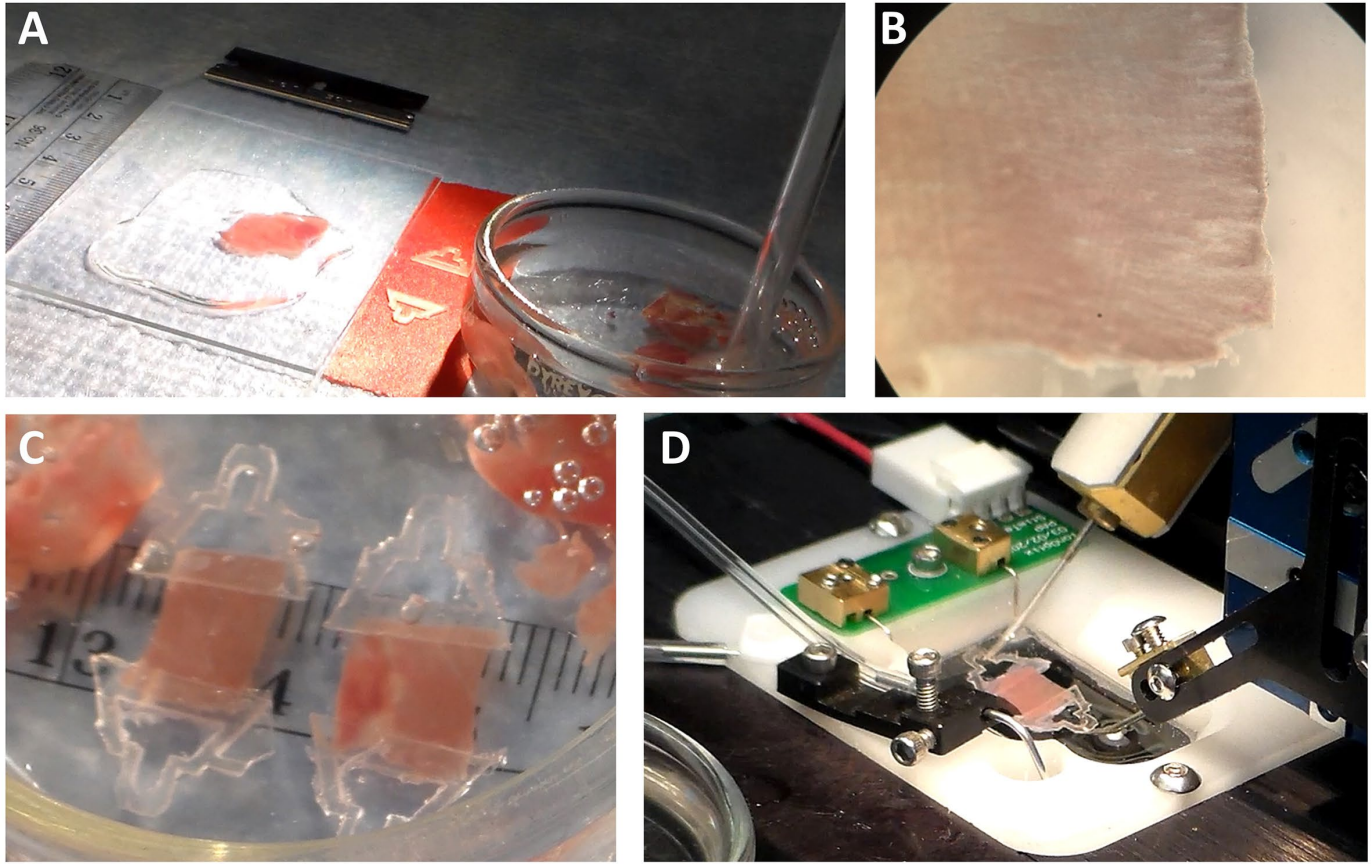
Clipping a Cardiac Slice. **(A)** The area required for clipping must be well lit surrounded by the necessary materials. **(B)** Fiber orientation must be detected using a dissection microscope. In this example, the fibers are running left to right. The lines running up and down are either creases from the cutting action of the blade and can be reduced with slower cutting speed. **(C)** Examples of clipped slices show the dimensions are roughly 10 mm long × 5–8 mm wide x 0.3 mm thick. **(D)** The clips permit the slice to be mounted into the recording apparatus.

Applying the clips to a cardiac slice requires a well-lit area, a dissection microscope, a larger glass slide, a new razor blade, a tube of cyanoacrylate adhesive (Histoacryl, Braun), a couple triangularly shaped clips, and two pairs of forceps (Figure 5A). Start by examining the slices through the microscope and choosing one that looks to have a fully intact and even surface.

Place a slice onto the glass and add oxygenated Dissection Solution to cover the slice. Gently flatten the slice onto the glass surface using forceps. Be careful not to pull on the muscle. Using the microscope, find the primary orientation of the muscle fibers in the slice. In the example of Figure 5B, the orientation is left to right. Using the razor blade, cut the tissue parallel and perpendicular with the orientation of the muscle fibers to produce a rectangle. The length of the rectangle running parallel with the fibers can be between 5 and 10 mm long. The width of the tissue can be between 5 and 10 mm. Once the rectangle has been made, remove excess solution from around the tissue. The tissue should be moist, but not wet, for the clip to be glued to the tissue.

Put some glue on a hard surface, such as another razor blade. Using forceps, take a clip and dip the broad end into the glue. Dab off excess glue and place it onto the end of the rectangularly shaped slice. Do the same for the other clip on the other end of the slice. After 20–40 s, the glue will adhere. Dissection Solution can then be reapplied to the tissue. Do not yet pick up a clip, as it may be adhered to the glass. First, push the tissue to assure none of the tissue is adhered to the glass surface prior to lifting it. Then return the tissue to the storage dish making sure it is submerged in the Dissection Solution (Figure 5C).

#### Mounting a Cardiac Slice

Carry the small dish with the clipped muscle the chamber. Adjust the distance between the platinum hooks using the manual micromanipulator so the hooks are slightly closer together than the distance between the ends of the clips. Also make sure the solution in the chamber is just covering the hooks and that the chamber is not full.

Pick up the muscle by one of the clips using forceps and carry it to the chamber. Put the end of a clip onto a hook attached to the length controller. Lay the tissue into the chamber to minimize the effects of surface tension, which will tend to force the tissue to the surface. Position the second clip over the other hook attached to the force transducer (Figure 5D). Once the tissue is in the chamber, use the manual micrometer to increase the distance between the hooks. Once there is some tension on the tissue, add additional solution to bring the chamber to a volume that covers the slice. A transfer pipette is useful to place solution on top of the slice in the chamber.

## Preparing to Record Functional Data

### Setup Before Recording

At this point, the muscle should be slack on the hooks. The Recording Solution should be bubbled with 100% O_2_ and can now be circulated through the chamber. The Recording Solution should be flowing at about 1–4 ml/min so that the chamber volume is exchanged every minute or two. The outflow from the chamber should go into waste for at least 5 min to assure that any residual Dissection Solution released from the muscle will be discarded. After those 5 min have passed, the Recording Solution can be recirculated to save solution volume.

The in-line solution heater can be turned on if a temperature higher than room temperature is desired. The new temperature will be stabilized within about 5 min of turning on the heating element. The aspirator height should be checked and adjusted if necessary to assure the muscle is submerged in the solution.

The stimulator should be set up to deliver a bipolar pulse to minimize electrolysis issues, between 1 and 5 ms in duration, and 1–100 V amplitude. Please note that the voltage amplitude of the stimulus will depend on the type of stimulator used. Some stimulators will activate muscle with as little as 1 V, while other stimulators may require as much as 30 V or more. This is because some instruments have low output impedance and cannot deliver sufficient stimulating current at low voltage. Start the voltage amplitude at a low value to assure that the threshold voltage will be discovered as stimulus amplitude is raised.

With the muscle loaded and no stimulation applied, adjust the length between the hooks to assure there is no tension on the muscle and the muscle is slack. Adjust the zero point of the force transducer so that zero force is recorded under this condition.

### Adjusting Stimulation Voltage and Frequency

Using the manual micromanipulator, slowly lengthen the distance between the hooks to assure there is some tension on the muscle. In the case of the papillary muscle, this will also assure there is a good electrical connection between the muscle and the stimulus. Turn on the stimulator in repeat mode. Starting with a low stimulating voltage, raise voltage to 1.5 times the stimulating threshold voltage. If the Dissection Solution is still washing out of the muscle, this washing out can often be visualized as an increase in twitch force for a few minutes as the BDM is removed from the muscle.

The stimulation frequency that allows for full relaxation between twitches can range from 0.2 to 10 Hz depending on the species, muscle type, and experimental conditions. For rat cardiac muscle at room temperature, a stimulation frequency of 1–3 Hz is reasonable. Human samples at room temperature function well at 0.2–1 Hz but may not be able to relax fully at higher frequencies up to 3 Hz, corresponding to 180 bpm, unless temperature is raised to 37°C. Samples from mouse heart at room temperature will function well at 1.5–3 Hz. Higher frequencies as high as 10 Hz can be achieved with mouse cardiac muscle at higher temperatures. Be aware that heart samples, especially from the smaller mammals, may spontaneously contract if the stimulation frequency is too low. In those cases, it is best to stimulate with a higher frequency that captures the muscle and gradually reduce stimulation frequency over 10–30 min until the muscle is stimulated at the desired frequency.

### Adjusting Starting Muscle Length

**Figure 6:**
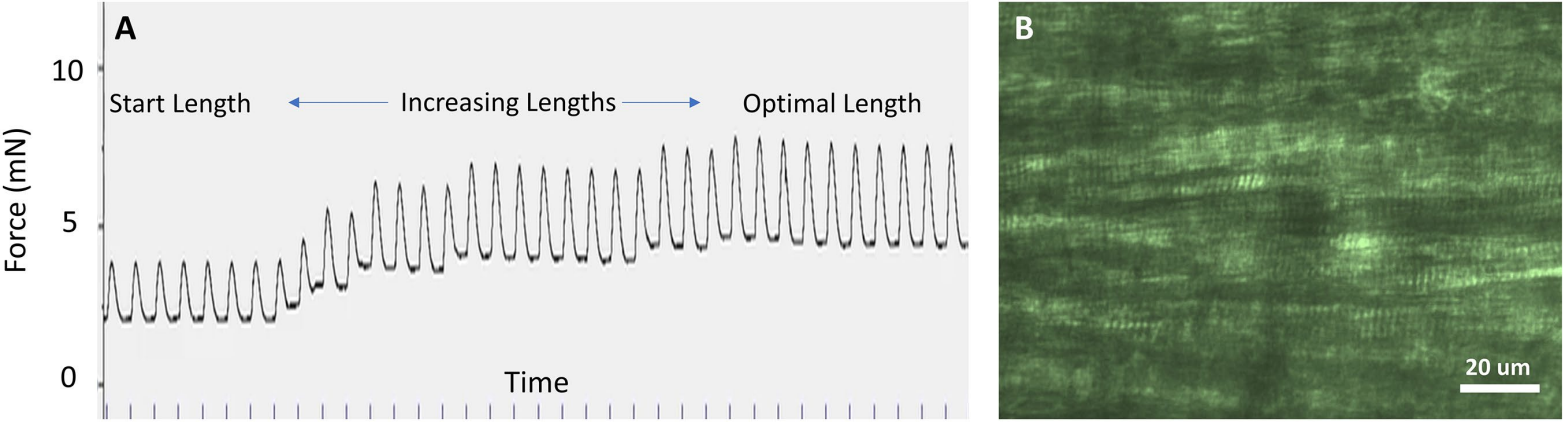
Starting muscle length. **(A)** When sarcomeres are not visible, the optimal muscle length can be determined by lengthening the muscle until the peak twitch force (systolic minus diastolic force) is maximized while the diastolic force is minimized. In this example, stimulation was repeated at 1 Hz. **(B)** In cardiac slices of ~0.2 mm thickness, sarcomeres are clearly visible using light microscopy and can be used to adjust sarcomere length. In this example, sarcomere length is 2.0 μm.

Once the stimulation voltage and frequency have been established and the Dissection Solution has washed out, the starting muscle length can be established. If the sarcomere pattern is not regularly visible, as is usually the case for papillary muscle, the initial muscle length can be established by stretching the muscle until the peak generated force is maximized. Starting from nearly slack muscle, the sarcomere length can be expected to be in the vicinity of 1.7–1.9 μm. As the muscle is lengthened, the sarcomere length will lengthen and systolic force will increase, which represents the ascending limb of the force–sarcomere length relationship. The optimal muscle length, often termed L_max_, can be found by lengthening the muscle until the highest developed force is achieved while maintaining the lowest possible diastolic force. In Figure 6A, the muscle is lengthened, and the developed force is found to rise with each incremental increase in length. This procedure should be repeated until there is no more rise in developed force with increasing length.

The optimal length coincides with sarcomere length approximately between 2.1 and 2.3 μm, which is the plateau region of the cardiac muscle force–sarcomere length relationship (de Tombe and ter Keurs, 2016). There are two factors that most influence the force–sarcomere length relationship and therefore optimal sarcomere length: (i) the fractional overlap of the thick and thin filaments, which is 100% within this range of sarcomere lengths, and (ii) whatever unidentified mechanism is further responsible for length-dependent activation in striated muscle (de Tombe and ter Keurs, 2016). The range of sarcomere lengths that corresponds to the optimal length can therefore be broad and will not correspond to a single sarcomere length for all preparations and conditions.

Sarcomeres are usually visible in cardiac slices thinner than ~250 μm (Figure 6B) and in skeletal muscle with a linear long axis, that is, not pennate. If possible, it is best to establish a sarcomere length by visualizing the sarcomere length using light microscopy as the initial step in the experimental procedure. We have found that diastolic sarcomere length can be routinely monitored in the cardiac slice, but systolic sarcomere length cannot be followed reliably. Fractional changes in whole muscle length are then in proportion to the fractional change in sarcomere length. Thus, establishing sarcomere length for a corresponding muscle length is an important initial step.

It should be noted that several attributes of the twitch force may change over time. The twitch force will generally rise slowly over the next hour and the diastolic force will generally hold steady or will slowly drop. If the diastolic force rises over time, it may be due to solution pH. We have found that even a temporary rise in pH will cause an irreversible rise in diastolic force. The changes in function that occur over time will depend upon species, muscle type, temperature, and stimulation frequency. The experimentalist must check to be sure that pH is maintained throughout the experiment.

## Materials and Equipment

Basic laboratory supplies must be available including glassware, double-distilled 18 MΩ water (ddH_2_O), refrigerator, pH meter, stir plate, ice, 100% O_2_ with regulator at 35 kPa (5 psi), tubing, and bubbling stones to deliver O_2_.

### Solutions

The solutions used in the preparation and examination of intact muscle are crucial to recording excitable muscle function. There are many solutions that have been used successfully. We provide here only one set of solutions that are useful for testing, demonstration, and practice. The user could use these solutions to answer many experimental questions, but ultimately must select solutions that will be most applicable for the physiological processes under investigation.

The Dissection Solution outlined in Table 1 is a HEPES-based, low Ca^2+^ Krebs-Ringer Solution useful for muscle preparation and not for recording muscle function. The Recording Solution outlined in Table 2 is a HEPES-based Krebs-Ringer Solution useful for functional analyses. Both solutions were proposed by Dr. Pieter de Tombe for use in investigating papillary muscles (de Tombe and Little, 1994). The use of HEPES rather than sodium bicarbonate as the buffering system makes it easier to maintain pH *in vitro*. However, be aware that many physiological processes rely upon bicarbonate, which is present *in vivo*.

**Table 1:** Dissection solution.

	2 L	1 L	500 ml	250 ml
NaCl (137 mM)	16.00 g	8.00 g	4.00 g	2.00 g
KCl (5.4 mM)	0.80 g	0.40 g	0.20 g	0.10 g
CaCl_2_ (0.2 mM)	0.40 ml	0.20 ml	0.10 ml	0.05 ml of 1 M Stock
MgCl_2_ (1.0 mM)	2.00 ml	1.00 ml	0.50 ml	0.25 ml of 1 M Stock
Glucose (10 mM)	3.60 g	1.80 g	0.90 g	0.45 g
HEPES (10 mM)	4.80 g	2.40 g	1.20 g	0.60 g
BDM (30 mM)	6.0 g	3.0 g	1.5 g	0.75 g

*This solution is useful for dissection and muscle preparation only and typically maintained cold ~ 4°C and oxygenated with 100% O_2_. Final concentrations are given in parentheses*.

**Table 2:** Recording solution.

	2 L	1 L	500 ml	250 ml
NaCl (137 mM)	16.00 g	8.00 g	4.00 g	2.00 g
KCl (4.5 mM)	0.68 g	0.34 g	0.17 g	0.09 g
CaCl_2_ (1.8 mM)	3.60 ml	1.80 ml	0.90 ml	0.45 ml of 1 M Stock
MgCl_2_ (1.0 mM)	2.00 ml	1.00 ml	0.50 ml	0.25 ml of 1 M Stock
Glucose (10 mM)	3.60 g	1.80 g	0.90 g	0.45 g
HEPES (10 mM)	4.80 g	2.40 g	1.20 g	0.60 g

*This solution is useful for recording function, such as twitch force, and can be used over a range of temperatures 15–40°C and must be oxygenated with 100% O*_2_.

The presence of 2,3-Butanedione Monoxime (BDM) in the Dissection Solution prevents muscle contraction during the dissection process and therefore preserves the muscle during preparation (Mulieri et al., 1989). The BDM will wash out of the muscle prior to recording function. Make the solutions according to the steps below.

1. Put ~90% of specified volume of ddH_2_O into a beaker or Erlenmeyer flask.
2. Add stir bar and stir.
3. Add each component listed in the table.
4. pH Dissection Solution to 7.35–7.40 at ~4°C (on ice) with NaOH.
5. pH Recording Solution to 7.35–7.40 at the temperature that will be used during recording with NaOH. Do not use KOH, which will add unwanted K^+^.
6. Take out the stir bar and pour into volumetric flask. Add ddH_2_O to final volume.
7. Filter and pour into clean, closable glass container.
8. Label (e.g., Krebs+HEPES+BDM or Krebs+HEPES+1.8Ca^2+^) and date
9. Close the container and store at 4°C.

When the solutions are needed for tissue dissection and data recording, be sure to bring the solutions to their respective temperatures, check pH and adjust if applicable, and bubble with 100% O_2_ prior to use. When long dissection periods are expected (e.g., >5 min), bubble with 100% O_2_ continuously. These solutions are best used fresh but can be kept at 4°C for up to 1 or 2 weeks.

#### Other Considerations Regarding Solutions

The presence of Ca^2+^ in the Recording Solution is essential for cardiac muscle, which requires extracellular Ca^2+^ to induce muscle contraction. The extracellular Ca^2+^ concentration can change depending upon the function under investigation and the species. Higher concentrations of extracellular Ca^2+^ (often reported up to 2.5 mM) will induce higher peak systolic force but can also induce higher diastolic force, which is undesirable. The experimentalist should be wary of this caveat and choose the extracellular Ca^2+^ concentration carefully.

In humans, the total plasma calcium is on the order of 2.2–2.6 mM and about half (1.1–1.3 mM) is in the form of ionic calcium (Pagana and Pagana, 2014). Anecdotally, the concentration of Ca^2+^ often appears in published studies to mimic more closely the total plasma calcium rather than ionic, for example, 2.4 mM in Runte et al. (2017). The range of total plasma calcium in mouse is dependent upon the mouse strain and encompasses 2.1–2.7 mM (Tordoff et al., 2007). The range of total plasma calcium for rats, rabbits, and other small mammals falls within the ranges of human and mouse, although the normal range for guinea pig can span 1.3–3.0 mM (Washington and Van Hoosier, 2012). The use of 1.8 mM Ca^2+^ as noted in Table 2 is a good starting point. Reasonable systolic and diastolic function would be expected, but this concentration should be adjusted according to the experimentalists needs and the physiology under investigation.

Those processes that require bicarbonate may be less physiological if using the HEPES-based solutions. For example, a HEPES-based solution resulted in a lower intracellular pH (~7.2) compared to a bicarbonate-HEPES solution (~7.3), and whole heart function was reduced (Imahashi et al., 2007). Bicarbonate can be added to the solution recipes in Tables 1 and 2, but sodium balance must be maintained. For example, the addition of 25 mM sodium bicarbonate (NaHCO₃) will require a concomitant reduction of 25 mM NaCl. And it will be necessary to bubble the solution with 95% O_2_-5% CO_2_ before and during the pH’ing process and to maintain pH during recording.

### Stations

Three stations that must be available: a gross dissection station, a muscle preparation station, and a recording station.

The gross dissection station is used to anesthetize and euthanize the animal subject and allows for removing the heart humanely. We will not provide information here about anesthetizing and euthanizing an animal or about removing the heart. Those procedures typically require training with a local veterinarian or physiologist, whose guidance will depend upon local laws and accepted practices of the experimentalist’s country, professional societies, and institution where the work is performed.

The muscle preparation station allows the experimentalist to remove the papillary muscles from the left ventricle and prepare the papillary muscles for recording and will be described below.

The recording station allows the recording of functional data from the papillary muscle and will also be described.

#### Muscle Preparation Station

**Figure 7:**
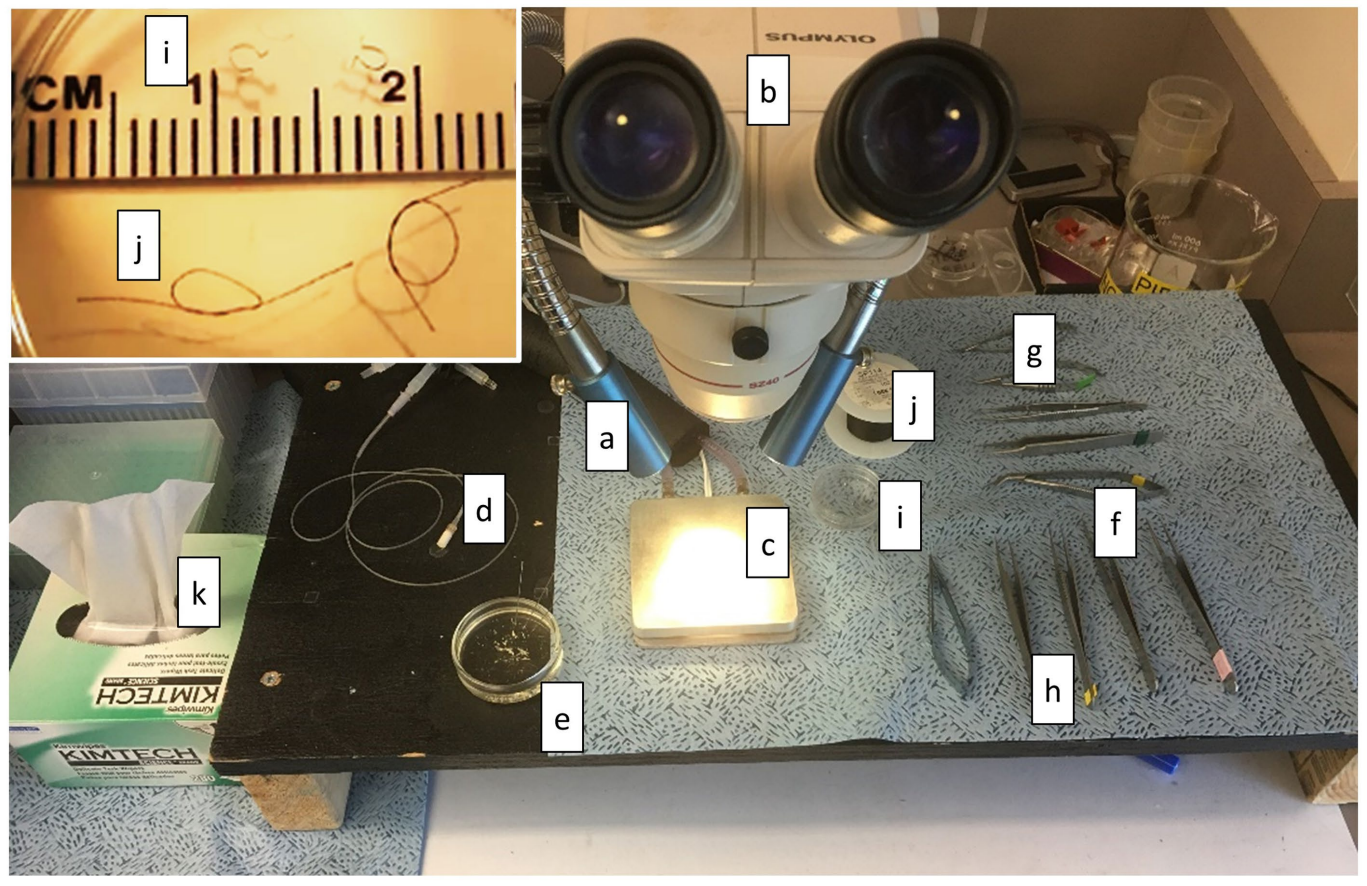
Muscle Preparation Station. The equipment and tools required to prepare muscle are shown here. (a) lighting, (b) dissection microscope, (c) cooling plate and circulator, (d) bubbling stone for delivering gases, (e) at least one shallow glass dish with a clear silicon or rubber bottom and dissection pins filled half way with Dissection Solution, (f) small scissors (~10–20 mm blade), (g) smaller scissors (~2–4 mm blade), (h) at least two pairs of forceps, (i) at least two platinum omega-shaped clips in a small glass or clear plastic dish (see inset), (j) 7-O silk suture with at least two double-thrown loops also in the dish (see inset), and (k) laboratory tissues.

The muscle preparation station must provide the following (Figure 7): (a) excellent lighting, (b) dissection microscope (10X magnification may be sufficient, but variable magnification up to 60X is preferable), (c) cooling plate and circulator (can be substituted with ice if a cooling plate and circulator are not available), (d) bubbling stone for delivering gases, (e) at least one shallow glass dish with a clear silicon or rubber bottom and dissection pins filled half way with Dissection Solution, (f) small scissors (~10–20 mm blade), (g) smaller scissors (~2–4 mm blade), (h) at least two pairs of forceps, (i) at least two platinum omega-shaped clips in a small glass or clear plastic dish, (j) 7-O silk suture with at least two double-thrown loops also in the dish, and (k) laboratory tissues. It is also important to have transfer pipettes, ice bucket, and additional Dissection Solution readily available.

#### Recording Station

**Figure 8:**
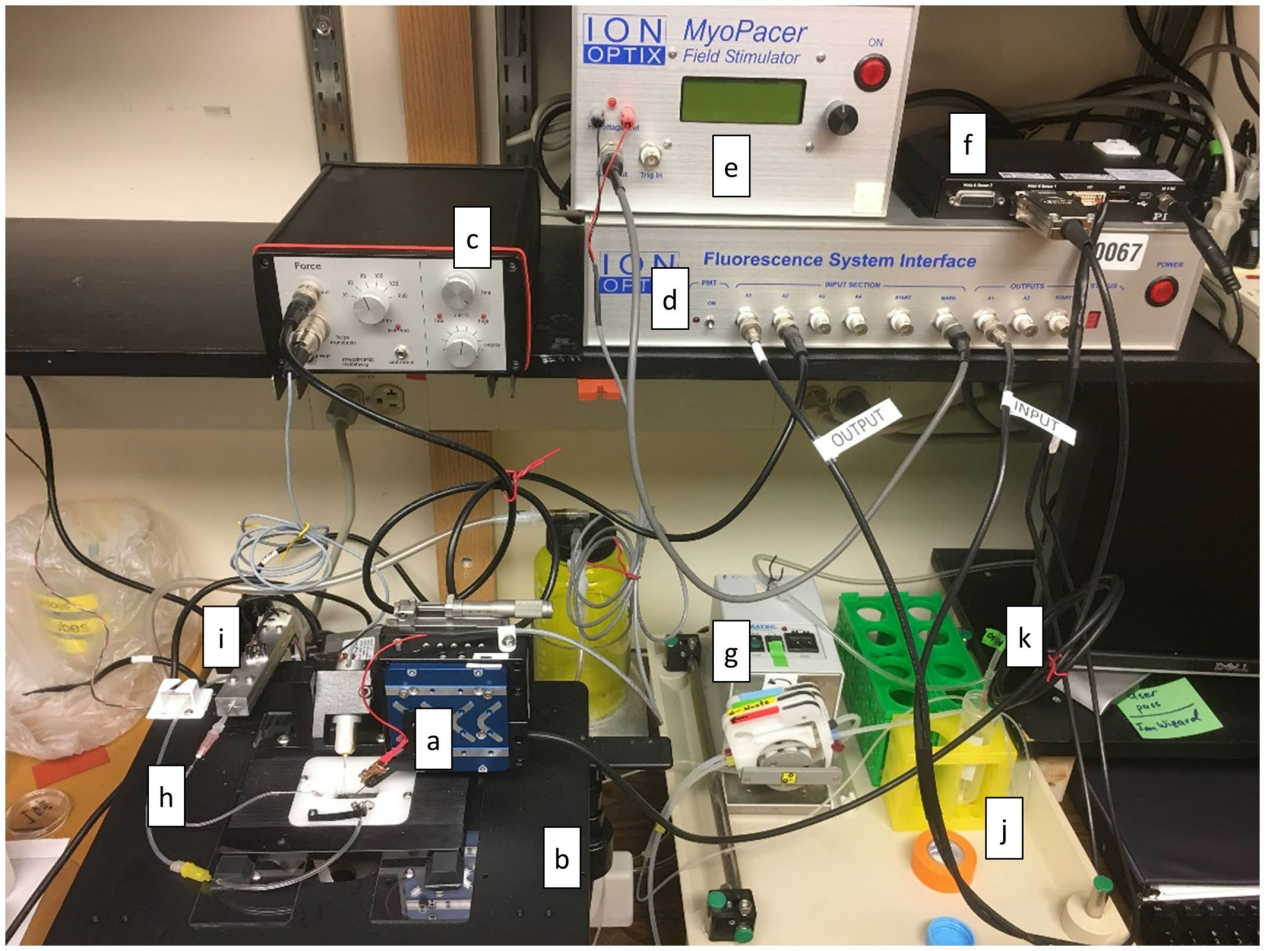
Recording Station. The equipment and tools required to record data associated with muscle function are shown here. (a) chamber fitted with force transducer and length adjuster, (b) inverted microscope with 20X, 25X, or 40X fluorescence objective, (c) bridge amplifier for force transducer, (d) electronic interface and computer required for data collection, (e) stimulator, (f) motor controller, (g) peristaltic circulating pump, (h) tubing for delivery and withdrawal of circulating solution, (i) in-line heater to warm the solution prior to delivery to chamber, (j) rack for holding beakers or tubes containing perfusion solutions, (k) bubbling stone to deliver gases to the perfusion solutions.

The recording station typically includes the following (Figure 8): (a) force transducer, length adjuster and chamber, (b) inverted microscope with 20X, 25X, or 40X fluorescence objective, (c) bridge amplifier for force transducer, (d) electronic interface and computer required for data collection, (e) stimulator, (f) motor controller, (g) peristaltic circulating pump, (h) tubing for delivery and withdrawal of circulating solution, (i) in-line heater to warm the solution prior to delivery to chamber, (j) rack for holding beakers or tubes containing perfusion solutions, (k) bubbling stone to deliver gases to the perfusion solutions. It is also important to have transfer pipettes and additional Recording Solution available. It is useful to have magnifying glasses, sometimes called jeweler’s magnifiers, for loading muscle into the chamber. Standard 1.75X magnification with 35 mm (14 in) focal length is recommended.

## Discussion

The cardiac papillary muscle has been studied for decades and represents a staple experimental model system for studying cardiac muscle physiology (de Tombe and Little, 1994). Similar linear preparations, such as cardiac trabeculae or strips, can be examined using similar methods provided the tissue consists of cells oriented in parallel along the long axis of the preparation (Chung et al., 2017; Runte et al., 2017). The cardiac slice is relatively new, at least in its widespread adoption (Pitoulis et al., 2020), and represents a robust model system that can remain viable for weeks (Fischer et al., 2019; Moretti et al., 2020). Both types of model systems, linear preparations and cardiac slices, can be subjected to length changes that mimic the cardiac pressure–volume relationship (Taberner et al., 2011). Furthermore, due to its longevity, the cardiac slice can be subjected to a specified pressure–volume profile long enough to undergo cardiac remodeling that mimics the *in vivo* response to pressure overload or volume overload (Pitoulis et al., 2021). The cardiac slice, again due to its longevity, can also support transfections of novel or mutant proteins. In these ways, the cardiac slice offers some advantages over linear preparations and isolated cardiac myocytes as a versatile experimental model system. The cardiac slice also offers routine visualization of sarcomere length, which is not possible in papillary muscles or whole hearts. It is likely that the cardiac slice will become the model system of choice as more laboratories begin to take advantage of its broad utility and relative ease of preparation (Watson et al., 2017).

Both the linear preparations and cardiac slices can be loaded with fluorescent dyes to examine calcium regulation (Runte et al., 2017) or action potential (Pitoulis et al., 2020). Action potentials over an entire cardiac slice can also be recorded with an array of microelectrodes (Halbach et al., 2006). With the various methods and assays available using the model systems presented here, several physiological phenomena could be explored. For example, the effects of sarcomere length on calcium dynamics or action potential can be examined; the effects of extracellular inorganic phosphate on cardiac function can be examined; the effects of protein mutations on length-dependent force activation can be examined in an intact muscle sample. These are just a few examples of the many possible experiments that could be performed with these models. We anticipate that several more physiological questions, protocols, and experimental measurement tools will arise as these model systems become more widely employed.

### Limitations of These Model Systems

While the linear preparation and the cardiac slice have much to offer, there are a few limitations the experimentalist should be aware of. For example, recording fluorescence from these preparations will often be accompanied by motion artifact. In the case of isolated myocytes, the field of view and depth of field can be chosen to encompass the entire myocyte, and total fluorescence from a single myocyte is negligibly affected by its motion. This is not the case in the more macroscopic preparations, although reasonably useful fluorescence signals are possible if care is taken to physically stabilize the sample in the field of view.

Papillary muscle and other linear preparations can experience a lack of metabolites at their core due to diffusion limitations. This phenomenon can hinder or prevent muscle performance when metabolite demands are high (Han et al., 2011). For this reason, linear preparations on the order of 200 mm diameter or smaller are required when high function and high stimulation rates are expected (Han et al., 2011). Again, the cardiac slice with a thickness under 250 μm will mitigate much of the issue related to diffusion limits.

### Conclusion

With the growing use of molecular methods to probe muscle physiology at the protein and sub-protein levels, there is a generation of muscle physiologists without much if any first-hand experience collecting function data at a level higher than the molecular. Yet the methods and skills required to collect functional data from macroscopic muscle preparations, like the papillary muscle or cardiac slice, are well established, clearly valuable, and still accessible to anyone willing to try. Much of the value in examining the macroscopic preparation stems from the preservation of *in vivo* structures that are lost or modified when single myocytes or molecules are isolated. The data collected with these model systems make for relatively easy translation to whole heart function and should be considered important components of assessing cardiac muscle function due to protein modification or response to drugs. With that need in mind, we have provided here some of the most basic methods for cardiac muscle preparation that are suitable for beginners and intermediate experimental physiologists.

## Data Availability Statement

The original contributions presented in the study are included in the article/supplementary material, further inquiries can be directed to the corresponding author.

## Ethics Statement

The animal study was reviewed and approved by University of Vermont.

## Author Contributions

BP and SB contributed to develop the methods presented in this article and revised the manuscript, read, and approved the submitted version. BP wrote the first draft of the manuscript. SB contributed the figures. All authors contributed to the article and approved the submitted version.

## Funding

This work was supported by National Institutes of Health (R44HL137603, BP) and by National Science Foundation (1660908, BP). Confocal microscopy was performed at the Microscopy Imaging Center at the University of Vermont (RRID# SCR_018821) using a Nikon A1R-ER point scanning confocal microscope supported by NIH award number 1S10OD025030-01 from the National Center for Research Resources. The authors are grateful for the expert technical assistance of Nicole Bouffard.

## Conflict of Interest

BP is employed by IonOptix, a manufacturer of equipment and software used in examine cardiac myocytes and small intact muscle preparations.

The remaining author declares that the research was conducted in the absence of any commercial or financial relationships that could be construed as a potential conflict of interest.

## Publisher’s Note

All claims expressed in this article are solely those of the authors and do not necessarily represent those of their affiliated organizations, or those of the publisher, the editors and the reviewers. Any product that may be evaluated in this article, or claim that may be made by its manufacturer, is not guaranteed or endorsed by the publisher.

## Footnotes

^1^ https://www.youtube.com/watch?v=VBaOjrf0NlU

^2^ https://www.youtube.com/watch?v=-ktp0mMNj1g

^3^ https://www.youtube.com/watch?v=3WcKLTUFPfw
